# Supplementary material for: SETDB1 regulates short interspersed nuclear elements and chromatin loop organization in mouse neural precursor cells
Source: Genome Biol. 2024 Jul 3;25:175. doi: 10.1186/s13059-024-03327-2 (PMC11221086; doi:10.1186/s13059-024-03327-2)
Supplement: Supplementary file 2 — Additional file 2: Fig. S1. Increased chromatin accessibility on SINE_B2 elements after Setdb1 ablation in mouse neural precursor cells. Fig. S2. Reduction of H3K9me3 on SINE_B2 after Setdb1 ablation in NPCs. Fig. S3. Alterations of DNA methylation after Setdb1 ablation in NPCs. Fig. S4. Increase of CTCF binding on SINE_B2 after Setdb1 ablation in NPCs. Fig. S5. Reorganization of chromatin loops on SINE_B2 with increased CTCF binding after Setdb1 ablation in NPCs. Fig. S6. Differential gene expression associated with loop reorganization after Setdb1 ablation. Fig. S7. Compromised NPC proliferation after Setdb1 ablation. [file 13059_2024_3327_MOESM2_ESM.pdf]

Sun D et al. SETDB1 regulates short interspersed nuclear elements and chromatin loop organization in mouse neural precursor cells

Additional file 2: Fig. S1-7.

Fig. S1. Increased chromatin accessibility on SINE_B2 elements after *Setdb1* ablation in mouse neural precursor cells.

Fig. S2. Reduction of H3K9me3 on SINE_B2 after *Setdb1* ablation in NPCs.

Fig. S3. Alterations of DNA methylation after *Setdb1* ablation in NPCs.

Fig. S4. Increase of CTCF binding on SINE_B2 after *Setdb1* ablation in NPCs.

Fig. S5. Reorganization of chromatin loops on SINE_B2 with increased CTCF binding after *Setdb1* ablation in NPCs.

Fig. S6. Differential gene expression associated with loop reorganization after *Setdb1* ablation.

Fig. S7. Compromised NPC proliferation after *Setdb1* ablation.

Fig. S1

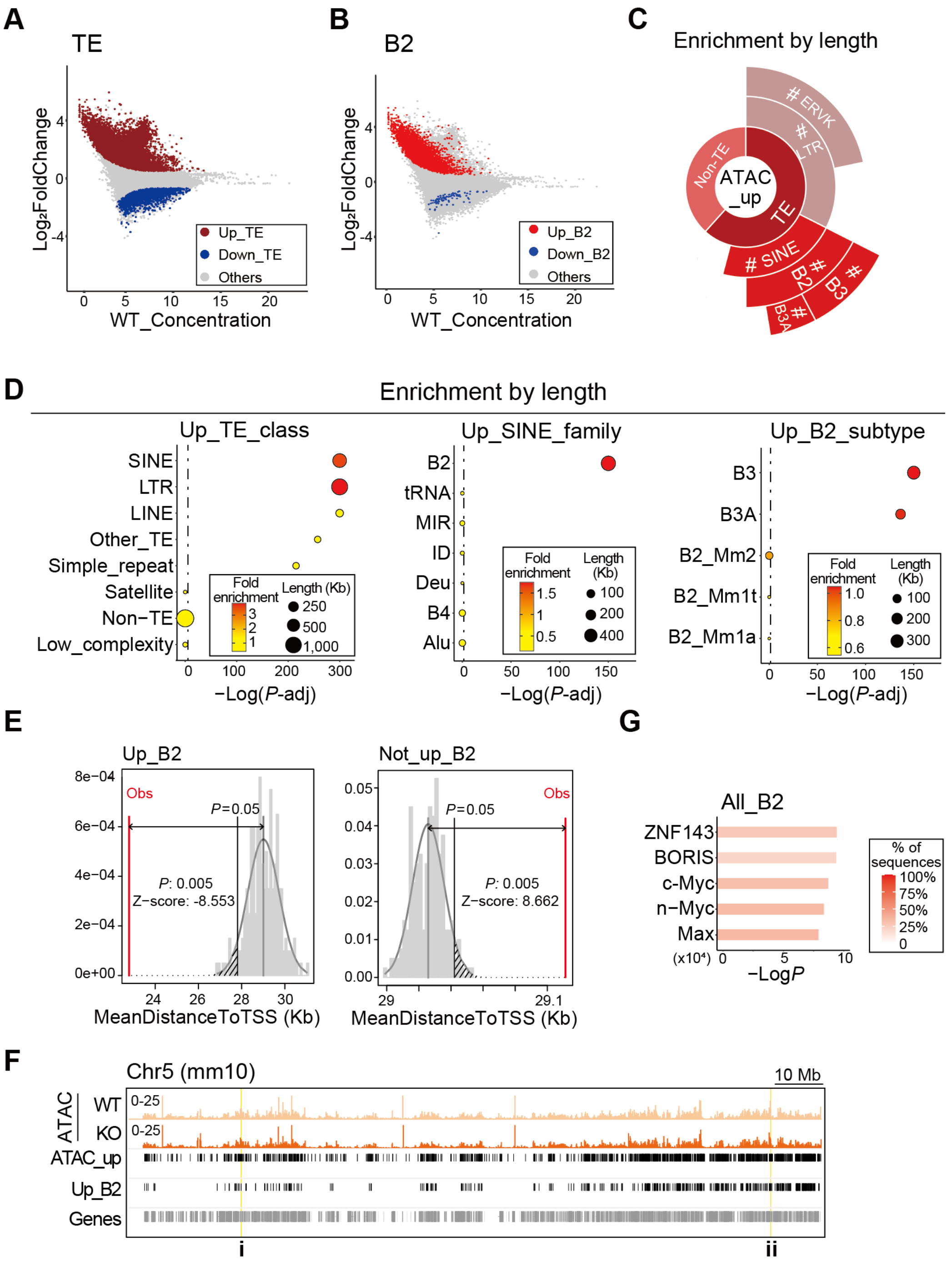

Fig. S1: Increased chromatin accessibility on SINE_B2 elements after *Setdb1* ablation in mouse neural precursor cells. (A-B) MA plots show differential ATAC-seq peaks annotated as TE **(A)** and SINE_B2 **(B)** (overlapping ≥ 50%). TE, transposable element. Red, Up. Blue, Down. Grey, Others. **(C)** Enrichment of up-regulated ATAC-seq peaks (ATAC_up) by accumulative length. **(D)** Enrichment of ATAC_up on “TE_class” (left),

"SINE_family" (middle) and "B2_subtype" (right) by accumulative length. Dotted lines indicate *P*-adj = 0.05. Fisher's exact test, B-H adjusted, $^{\#}P < 0.0001$. **(E)** Permutation tests of the mean distance to TSS from B2 overlapping (Up_B2, left) or not overlapping ATAC_up (Not_up_B2, right). TSS, transcription start site. Black, *P* = 0.05. Red, observed (Obs). **(F)** IGV map tracks show ATAC signal of WT and KO on Chr5. Vertical bars indicate sites of ATAC_up and Up_B2. Yellow shades indicate zoomed-in regions **i** and **ii** in the main **Fig. 1G**. **(G)** Top 5 enriched Homer known motifs of all B2 elements (All_B2).

Fig. S2

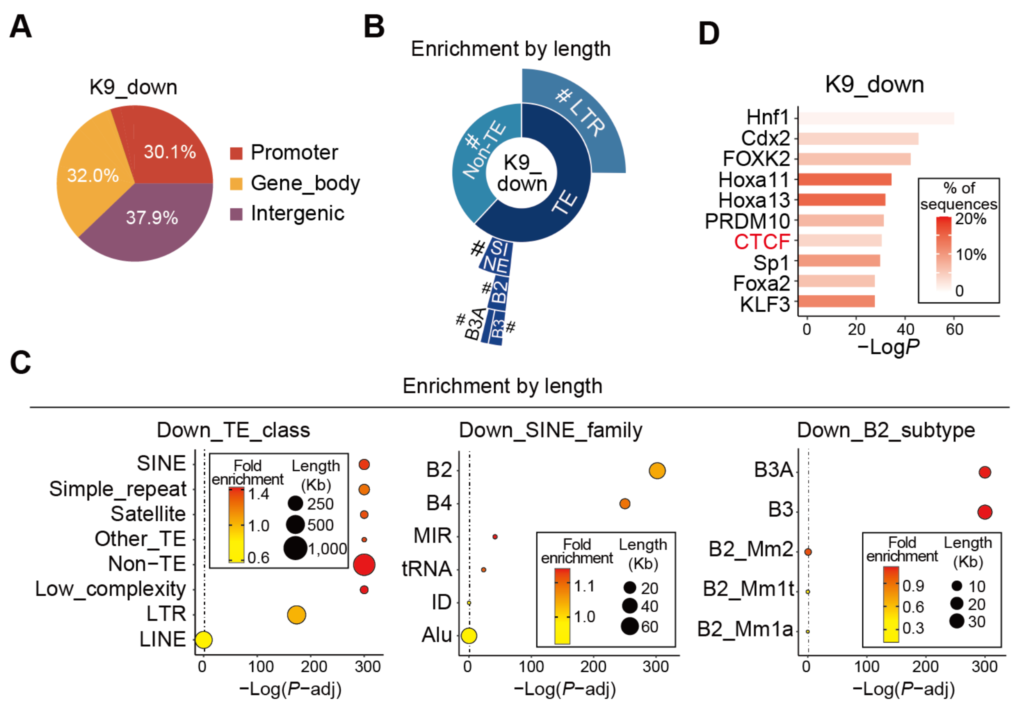

Fig. S2: Reduction of H3K9me3 on SINE_B2 after *Setdb1* ablation in NPCs. **(A)** Genomic distribution of significantly down-regulated H3K9me3 ChIP-seq peaks (K9_down). Red, Promoter. Yellow, Gene_body. Purple, Intergenic. **(B)** Enrichment of K9_down by accumulative length. **(C)** Enrichment of K9_down on “TE_class” (left), “SINE_family” (middle) and “B2_subtype” (right) by accumulative length. Dotted lines indicate *P*-adj = 0.05. Fisher’s exact test, B-H adjusted, $^{\#}P < 0.0001$. **(D)** Top 10 enriched Homer known motifs of K9_down. Note that CTCF motif (red) was enriched.

Fig. S3

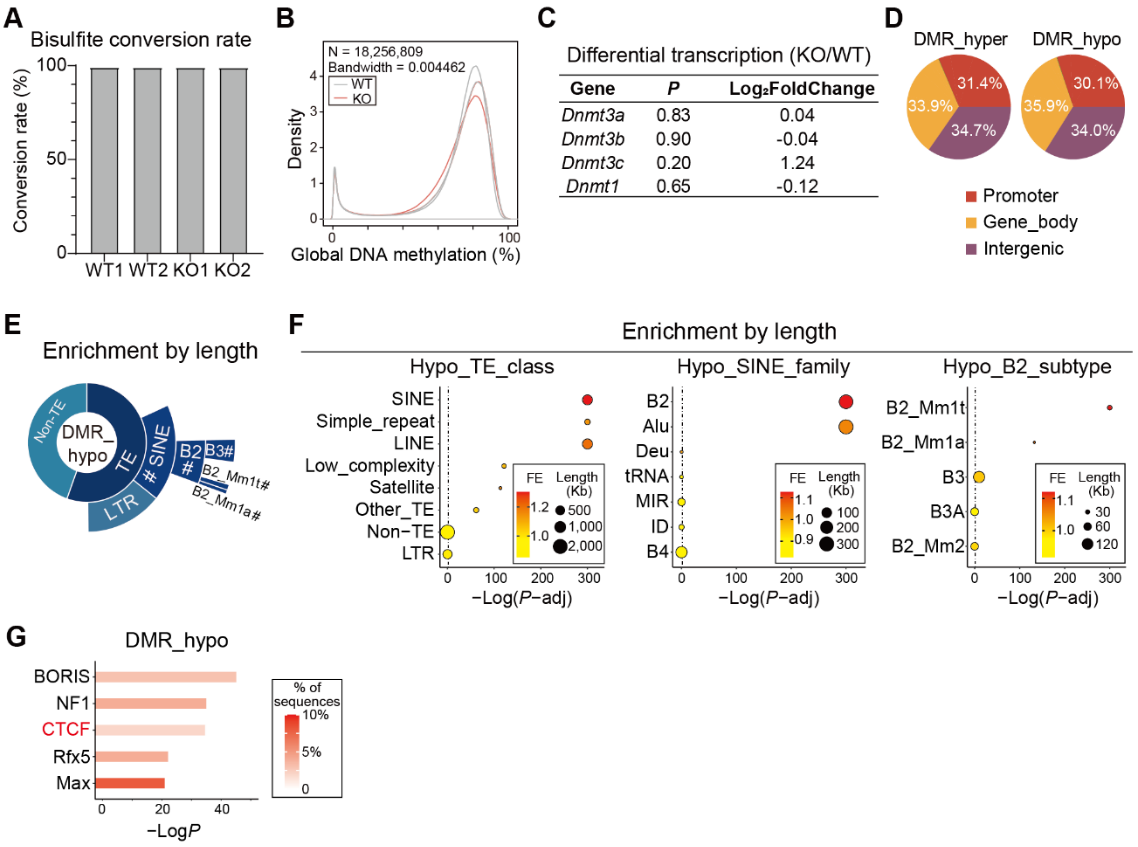

Fig. S3: Alterations of DNA methylation after *Setdb1* ablation in NPCs. (A) Bisulfite conversion rate of WGBS samples. **(B)** Density plot displays global DNA methylation level of WT (grey) and KO (red) NPC. **(C)** Differential transcription analysis (RNA-seq) of detected genes encoding DNA methyltransferases. KO/WT, N = 3. **(D)** Genomic distribution of significantly hypermethylated (DMR_hyper, left) and hypomethylated (DMR_hypo, right) regions. Red, Promoter. Yellow, Gene_body. Purple, Intergenic. **(E)** Enrichment of DMR_hypo by accumulative length. **(F)** Enrichment of DMR_hypo on “TE_class” (left), “SINE_family” (middle) and “B2_subtype” (right) by accumulative length. FE, fold enrichment. Dotted lines indicate *P*-adj = 0.05. Fisher’s exact test, B-H adjusted, $^{\#}P < 0.0001$. **(G)** Top 5 enriched Homer known motifs of DMR_hypo. Note CTCF motif (red) was enriched.

Fig. S4

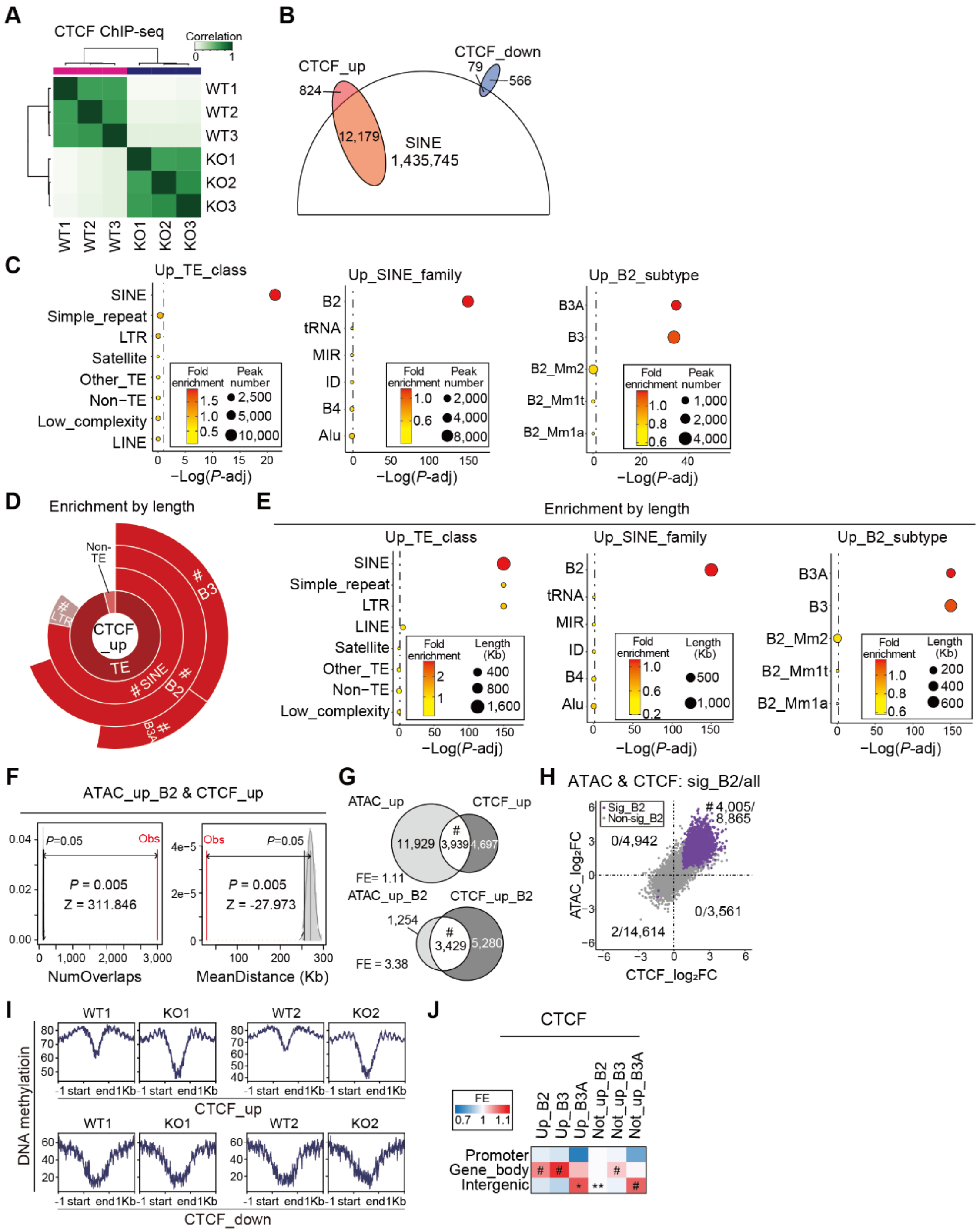

Fig. S4: Increase of CTCF binding on SINE_B2 after *Setdb1* ablation in NPCs. (A) Correlation heatmap of CTCF ChIP-seq samples derived from WT and KO NPCs. **(B)** Venn diagram displays the overlap of SINE elements with up-regulated (CTCF_up, red) and down-regulated CTCF peaks (CTCF_down, blue). **(C)** Enrichment of CTCF_up peaks

on “TE_class” (left), “SINE_family” (middle) and “B2_subtype” (right) by number. Dotted lines indicate P-adj = 0.05. **(D)** Enrichment of CTCF_up by accumulative length. **(E)** Enrichment of CTCF_up on “TE_class” (left), “SINE_family” (middle) and “B2_name” (right) by accumulative length. Dotted lines indicate P-adj = 0.05. **(F)** Permutation tests on the number of overlaps (NumOverlaps) between ATAC_up_B2 and CTCF_up (left), and the mean distance from ATAC_up_B2 to CTCF_up (right). Black, $P = 0.05$. Red, observed (Obs). **(G)** Venn diagrams display the overlap between ATAC_up and CTCF_up (top), as well as ATAC_up_B2 and CTCF_up_B2 (bottom). FE, fold enrichment. **(H)** Log_2FoldChange (log_2FC) for overlapping ATAC and CTCF peaks. sig_B2/all, significantly differential ATAC and CTCF peaks on B2 elements/all overlapping ATAC and CTCF peaks. Purple, sig_B2. Grey, non-sig_B2. Note sig_B2 is enriched in the 1st quadrant. **(I)** Signal profiles of DNA methylation on CTCF_up (top) and CTCF_down peaks (bottom). **(J)** Genomic distribution of CTCF_up_B2, CTCF_up_B3, CTCF_up_B3A, CTCF_not_up_B2, CTCF_not_up_B3 and CTCF_not_up_B3A. Fisher’s exact test, B-H adjusted, $^{*}P < 0.05$, $^{**}P < 0.01$, $^{***}P < 0.001$, $^{\#}P < 0.0001$.

Fig. S5

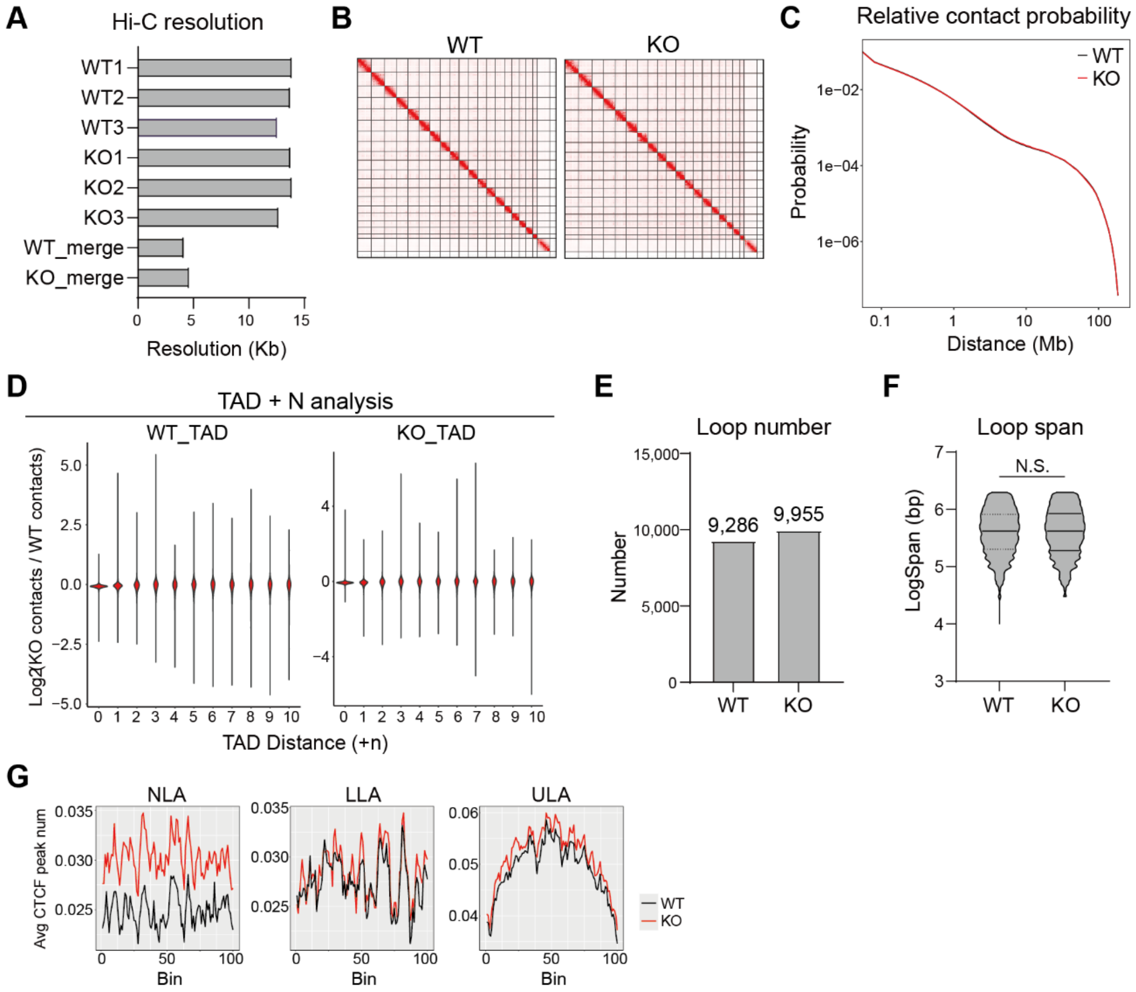

Fig. S5: Reorganization of chromatin loops on SINE_B2 with increased CTCF binding after *Setdb1* ablation in NPCs. (A) Hi-C contact map resolution of individual (N = 3 WT/3 KO) and merged samples by genotype. **(B)** Hi-C contact maps of WT and KO NPC. **(C)** Genome-wide relative contact probability in WT (black) and KO (red). **(D)** Differential (KO/WT) contact density between neighboring TADs in WT (left) and KO (right) TAD regions. **(E-F)** The number **(E)** and span **(F)** of chromatin loops in WT and KO. N = 9,268 WT/9,955 KO. Two-tailed Mann-Whitney *U* test, N.S., non-significant. **(G)** Average CTCF ChIP-seq peak numbers on “new_loop_anchor” (NLA, left), “lost_loop_anchor” (LLA, middle), and “unchanged_loop_anchor” (ULA, right). Black, WT; red, KO.

Fig. S6

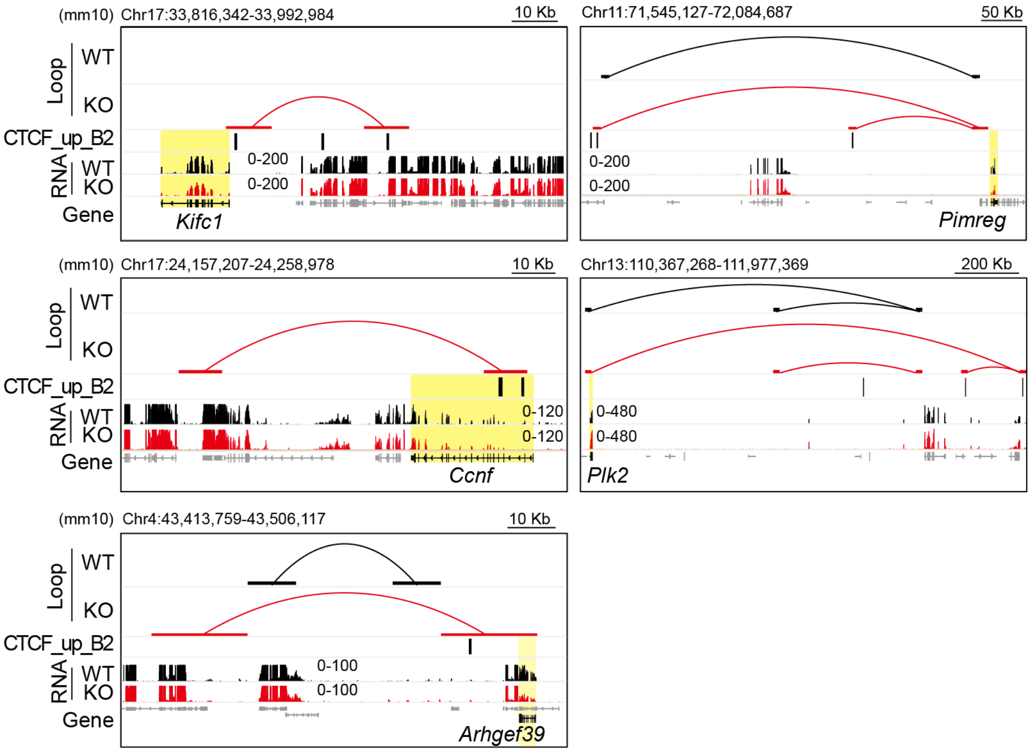

Fig. S6: Differential gene expression associated with loop reorganization after *Setdb1* ablation. IGV map tracks show examples of differentially expressed genes (DEGs) anchored by New_loops on B2 elements (B2_new_loops). Yellow shade, DEGs anchored by B2_new_loops.

Fig. S7

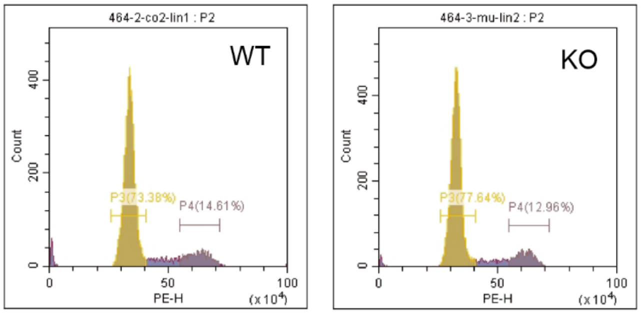

Fig. S7: Compromised NPC proliferation after *Setdb1* ablation. Representative flow cytometry images for NPC cell cycle analysis.
