# Supplementary material for: SETDB1 regulates short interspersed nuclear elements and chromatin loop organization in mouse neural precursor cells
Source: Genome Biol. 2024 Jul 3;25:175. doi: 10.1186/s13059-024-03327-2 (PMC11221086; doi:10.1186/s13059-024-03327-2)
Supplement: Supplementary file 3 — Additional file 3. Review history. [file 13059_2024_3327_MOESM3_ESM.docx]

Review History

**First round of review**

**Reviewer 1**

**Were you able to assess all statistics in the manuscript, including the appropriateness of statistical tests used?**

Yes, and I have assessed the statistics in my report.

**Comments to author:**

This manuscript employs ATAC-seq, ChIP-seq, WGBS, in situ Hi-C, and RNA-seq in mouse NPCs to elucidate the mechanism by which SETDB1 regulates SINEs. The story is interesting and the data quality of the manuscript is high. Upon addressing the following issues, the manuscript can be considered for publication.

Major points:

1.Whether in the abstract (Line 44-45) or the introduction (Line 82-83), it appears that the authors aim to address the role of SINEs in neurodevelopmental processes. However, throughout the manuscript, the authors did not investigate SINEs but instead focused on studying the impact of SETDB1. This seems to be inconsistent with the scientific question. Please consider revising the statement or adding experiments to assess the effect of knocking out SINEs on NPCs.

2.The authors consistently describe the effects of SETDB1 knockout throughout the manuscript. SETDB1 is known to have both enzymatic and non-enzymatic functions. After knocking out SETDB1, it's worth investigating whether the non-enzymatic functions of SETDB1 are affected. If it is possible to supplement enzymatic mutant variants, that would be ideal. However, if this is not feasible, it is advisable to discuss the authors' viewpoint in the Discussion section, acknowledging the importance of exploring the impact of SETDB1 knockout on its non-enzymatic functions as an alternative approach.

3.The analysis between Figure 4 and Figure 5 appears disjointed. While Line288-300 demonstrates that CTCF regulates 3D chromatin architecture, the analysis of differential loops in Figure 5 lacks correlation with the differential binding of CTCF. Are all these differential loops mediated by CTCF? It is important to perform an integrated analysis by combining Hi-C data with CTCF ChIP data and considering the orientation of CTCF motifs (convergent motif orientation at both ends of CTCF-mediated loops).

4.The differential gene analysis in Figure 6 should be combined with ATAC-seq, H3K9me3 ChIP-seq, CTCF ChIP-seq, and WGBS to perform joint analysis and identify shared differential regions，at least give an example.

Minor points
1.Line 280-286, I cannot understand why the authors described "H3K9me3 and DNA methylation play together, but with different ways" when it is apparent that both of them decrease after SETDB1 knockout. Additionally, at Line 283-284, this phenomenon is quite common. Regions bound by CTCF are typically DNA methylation valleys, which are areas of low methylation. Therefore, it is common for their signals to be lower than the background.

2.Line 292-295, Does B2 typically occur in the promoter region? If it doesn't naturally occur in the promoter region, it seems normal for CTCF_up peaks on the B2 not to be in the promoter region.

3.The viewpoint aligns with Major Point 3, necessitating an analysis of the number of differential loops and how many of them are mediated by CTCF. The analysis of 5C is deemed meaningless, as any manipulation could yield the same result. Instead, it is advisable to analyze the overall change in loop intensity.

4.Line 325, What are "major 3D genome structures"?

5.Lines 336, Avoid using the word "novel."

6.Line392-393, The full name of ESC has already been mentioned above and doesn't need to be described again.

7.Line 816 has an extra line break.

**Reviewer 2**

**Were you able to assess all statistics in the manuscript, including the appropriateness of statistical tests used?**

No, I do not feel adequately qualified to assess the statistics.

**Comments to author:**

This is an exciting paper describing how genetic ablation of Kmt1e/Eset/Setdb1 methyltransferase in neural precursor cells causes a epigenomic (though not necessarily transcriptional) repression of a subset of SINE elements, which then engage in de novo loop formations leading to de-repression and dysregulation of mitosis genes. The authors validate the functional implications, decreased proliferation, with in vitro and vivo assays.
Mechanistically, they show that de novo/excess CTCF bindings occurs at these subset of SINEs.

This is certainly an impactful study. My main comment is that the authors undersell a bit their main findings, the abstract and even the results and discussion section seem to put the emphasis more on other things reported in the paper. I recommend to refocus /rewrite a bit.

Other comments:
a) how many loops are formed de novo ?
b) how many mitosis genes are dysregulated. May be both these questions are addressed in the manuscript but reading through it I either missed it or teh authors did not emphasize well.

c) is there excess CTCF binding at the loop end where the mitosis gene is located?
d) the authors mapped DNA methylation genome-wide, but whether CTCF motifs / CTCF bindign sites are hypomethylated in their mutatn cells is not addressed (unless I missed it) . Loss of DNA methylation at CTCF motif sites could explain excess CTCF binding as DNA methylation inhibits CTCF docking to the DNA, if I am informed correctly?

e) are all dividing cells show these dysregulation of mitosis genes and proliferation phenotype after setdb1 ablation? or is this unique to NPC?
f) the authors show evidence for decreased proliferation . is this just a slowing of cell cycle, meaning the NPC still divide but the mitosis cycle takes more time/decreased duplication rate, or is there a block in the cell cycle.
g) what makes SINEs interacting with mitosis genes unique? any specific sequence signature or transcription factor motifs? and how many Sine elements are dysregulated in teh mutant, is it possible to estimate this.

**Authors Response**

**Point-by-point responses to the reviewers’ comments:**

Reviewer #1:

Major comments:

1. “Whether in the abstract (Line 44-45) or the introduction (Line 82-83), it appears that the authors aim to address the role of SINEs in neurodevelopmental processes. However, throughout the manuscript, the authors did not investigate SINEs but instead focused on studying the impact of SETDB1. This seems to be inconsistent with the scientific question. Please consider revising the statement or adding experiments to assess the effect of knocking out SINEs on NPCs.”

***Response:*** *Thank you for your suggestions. We have revised our statements to address the regulatory mechanisms controlling the activity of SINEs in the developing brain. (****Line 44-46****,* ***Line 83-84****)*

2. “The authors consistently describe the effects of SETDB1 knockout throughout the manuscript. SETDB1 is known to have both enzymatic and non-enzymatic functions. After knocking out SETDB1, it's worth investigating whether the non-enzymatic functions of SETDB1 are affected. If it is possible to supplement enzymatic mutant variants, that would be ideal. However, if this is not feasible, it is advisable to discuss the authors' viewpoint in the Discussion section, acknowledging the importance of exploring the impact of SETDB1 knockout on its non-enzymatic functions as an alternative approach.”

***Response:*** *Thank you for your suggestion. As advised,* *we have added a new paragraph in the Discussion section. In the literature, a truncated splicing isoform of SETDB1, devoid of catalytic SET domain, was detected in the mouse brain. Moreover, the non-enzymatic functions of SETDB1 were reported in regulating gene expression and genome organization (2022, Warrier T et al., Nucleic Acids Res; 2015, Fei Q et al., Genome Res). In our study, the knockout strategy generates a premature stop codon right after the original exon 2 of Setdb1, which causes the loss of all the major functional domains and disrupts* *both enzymatic and non-enzymatic functions. Although our current study focused on SETDB1-mediated H3K9me3 repression on SINE_B2 activity, we cannot rule out the contribution of its non-enzymatic role in regulating DNA repeats including SINEs, thus would be important for future investigation. (****Line 416-432****)*

3. “The analysis between Figure 4 and Figure 5 appears disjointed. While Line288-300 demonstrates that CTCF regulates 3D chromatin architecture, the analysis of differential loops in Figure 5 lacks correlation with the differential binding of CTCF. Are all these differential loops mediated by CTCF? It is important to perform an integrated analysis by combining Hi-C data with CTCF ChIP data and considering the orientation of CTCF motifs (convergent motif orientation at both ends of CTCF-mediated loops).”

**Response:** Thank you for your suggestions. We have performed integrated analysis of Hi-C and CTCF ChIP-seq to demonstrate that the de novo loops in KO were contributed by increased CTCF binding. Both CTCF signal (new **Fig. 5E**) and the number of CTCF peaks (new **Fig. S5G**) were increased on newly formed loop anchors (NLAs) in KO. Notably, a significant portion (1,195 out of 6,444) of NLAs (P < 0.0001, fold enrichment = 1.85) overlapped with increased CTCF peaks on B2 elements (new **Fig. 5F**). Moreover, consistent with published data, most of these new loops were anchored on convergent CTCF binding motifs (~70%) (new **Fig. 5H**). We have now added these data in the Results section (**Line 325-331, 334-340**) and Methods section (**Line 737-743, 747-749**)

4. “The differential gene analysis in Figure 6 should be combined with ATAC-seq, H3K9me3 ChIP-seq, CTCF ChIP-seq, and WGBS to perform joint analysis and identify shared differential regions, at least give an example.”

***Response:*** *Thank you for your suggestion. As advised, we have performed joint analysis of ATAC-seq, H3K9me3 ChIP-seq, CTCF ChIP-seq, WGBS and RNA-seq. Increased chromatin accessibility, loss of H3K9me3, significant DNA hypomethylation, and increased CTCF bindings were observed on the population of CTCF_up_B2 elements associated with differentially expressed genes (DEGs) and new loops in KO (new* ***Fig. 6C****)****.*** *Additionally, we have provided an example to display the transcriptional and epigenetic signatures on one of the DEGs, Cenpe, and its associated new loop anchor (new* ***Fig. 6G****). We have now added these data in the Results section* *(****Line 359-361, 368-370****). Accordingly, we moved other examples for DEGs in the original* ***Fig. 6E*** *to the supplementary material as* ***Fig. S6.***

Minor comments:

1. “Line 280-286, I cannot understand why the authors described "H3K9me3 and DNA methylation play together, but with different ways" when it is apparent that both of them decrease after SETDB1 knockout. Additionally, at Line 283-284, this phenomenon is quite common. Regions bound by CTCF are typically DNA methylation valleys, which are areas of low methylation. Therefore, it is common for their signals to be lower than the background.”

***Response:*** *Thank you for your advice. We have revised our statement to “**These observations suggest that H3K9me3 and DNA methylation both contribute to the repression of CBS on SINE_B2 elements.” (****Line 293-294****)*

2. “Line 292-295, Does B2 typically occur in the promoter region? If it doesn't naturally occur in the promoter region, it seems normal for CTCF_up peaks on the B2 not to be in the promoter region.”

***Response:*** *Thank you for your careful review. As pointed out, B2 elements, especially the altered population in our study were primarily located in the gene bodies, which were in line with the observation that up-regulated CTCF binding almost exclusively occurred on non-promoter regions. We brought up this information to: 1) support that the increase of CTCF resulted from changes on B2 elements; 2) suggest the impact on gene transcription was not from direct changes of epigenetic signatures on the promoters, but could from indirect regulations such as CTCF-mediated chromatin interactions. This led to our subsequent studies on loop formation and differential gene expression. We have now changed the statements in the revised manuscript (****Line 300-304****).*

3. “The viewpoint aligns with Major Point 3, necessitating an analysis of the number of differential loops and how many of them are mediated by CTCF.”

***Response:*** *Thank you for your suggestions. As advised, we have presented the joint analysis of loops and CTCF binding in our response to Major Point 3.*

“The analysis of 5C is deemed meaningless, as any manipulation could yield the same result. Instead, it is advisable to analyze the overall change in loop intensity.”

***Response:*** *As advised, we have made changes in the original Fig. 5C to show overall change in loop intensity from WT and KO NPCs. (new* ***Fig. 5C****,* ***Line 321-322****)*

4. “Line 325, What are "major 3D genome structures"?”

***Response:*** *Thank you for your question. We have revised it to “the conformation of TADs” in the new manuscript. (****Line 343****)*

5. “Lines 336, Avoid using the word "novel."”

***Response:*** *As advised, we have changed to “newly formed”. (****Line 356****)*

6. “Line392-393, The full name of ESC has already been mentioned above and doesn't need to be described again.”

***Response:*** *Thank you for your kind notice. We have made the correction. (****Line 442****)*

7. “Line 816 has an extra line break.”

***Response:*** *We have carefully checked the format of references, and this now becomes the new* ***reference #51*** *in the new manuscript. (****Line 906-908****)*

Reviewer #2:

Main comment:

“My main comment is that the authors undersell a bit their main findings, the abstract and even the results and discussion section seem to put the emphasis more on other things reported in the paper. I recommend to refocus /rewrite a bit.”

***Response:*** *Thank you for your appreciation of our studies. We have now revised our manuscript as suggested with the emphasis on the regulatory role of SETDB1 on CTCF bindings at SINE elements. Moreover, CTCF-mediated de novo loop formations after the loss of Setdb1 led to the dysregulation of mitosis genes in NPCs, which contributed to the compromised cell proliferation both in vitro and in vivo.*

Other comments:

a) “How many loops are formed de novo?”

***Response:*** *In our study, 7,020 loops were formed de novo in Setdb1-knockout NPCs. (****Line 336****)*

b) “How many mitosis genes are dysregulated? Maybe both these questions are addressed in the manuscript but reading through it I either missed it or the authors did not emphasize well.”

***Response:*** *We apologize for missing these important information in our original manuscript. We have now added it to the revised manuscript that 120 mitosis genes were dysregulated in Setdb1-knockout NPCs. (****Line 351-353****)*

c) “Is there excess CTCF binding at the loop end where the mitosis gene is located?”

***Response:*** *Thank you for your question. In our study, excess CTCF binding was observed on the B2 element at the loop anchors that connected the differential mitosis genes in KO (new* ***Fig. 6C****). We also provide an example to illustrate epigenetic signatures including increased CTCF binding on the anchor of a loop connecting the mitosis gene Cenpe (new* ***Fig. 6G****). We have now added these data in the Results section.* *(****Line 359-361, 368-370****)*

d) “The authors mapped DNA methylation genome-wide, but whether CTCF motifs / CTCF binding sites are hypomethylated in their mutant cells is not addressed (unless I missed it). Loss of DNA methylation at CTCF motif sites could explain excess CTCF binding as DNA methylation inhibits CTCF docking to the DNA, if I am informed correctly?”

***Response:*** *Thank you for your suggestions. As advised, we have analysed DNA methylation on differential CTCF peaks, and observed loss of DNA methylation on the up-regulated CTCF binding sites (CTCF_up peaks) in KO (new* ***Fig. S4I****). We have now added these data in the Results section.* *(****Line 276-281****)*

e) “Are all dividing cells show these dysregulations of mitosis genes and proliferation phenotype after Setdb1 ablation? or is this unique to NPC?”

***Response:*** *Thank you for raising this important question. SETDB1 has been reported in the regulation of cell division and proliferation in multiple systems, including germ cells* *(2016, Eymery A et al., Development), skeletal system (2022, Zhang L et al., Cells) and cancer cells (2019, Wang G et al., Nat Cell Biol; 2016, Wong CM et al., Hepatology). However, this regulation has not been reported in NPCs before. Our study provide evidence and epigenetic mechanism for the role of SETDB1 in regulating NPC proliferation. We have included this information in the revised manuscript. (****Line 374-377****)*

f) “The authors show evidence for decreased proliferation. is this just a slowing of cell cycle, meaning the NPC still divide but the mitosis cycle takes more time/decreased duplication rate, or is there a block in the cell cycle.”

***Response:*** *Our results suggested that the cell cycle of KO NPCs was not totally blocked, as they still formed neurospheres, although with smaller size. In addition, a proportion of KO NPCs were still observed in the G2/M phase and positive for PH3 staining. However, the proportion of NPCs in G2/M phase was decreased, while those in G1 phase were increased in KO, suggesting a slowing of cell cycle after the loss of Setdb1. We now provide representative flow cytometry images for cell cycle analysis in both WT and KO (new* ***Fig. S7****). We have included this information in the revised manuscript. (****Line 379-381, 386-388****)*

g) “What makes SINEs interacting with mitosis genes unique? any specific sequence signature or transcription factor motifs?”

***Response:*** *Thank you for raising these important questions. We conducted Homer motif analysis on the promoters of differential mitosis genes that were associated with new loops. The top 5 enriched motifs were CHR (the homology region for cell cycle gene promoters that recruited regulatory DREAM complex), JunD, NRF1, Sp2 and Sp1 (new* ***Fig. 6F****). Interestingly, these transcription factors (TFs) were all reported as binding partners or co-regulatory factors of CTCF (2014, Bohla D et al., PLoS One; 2021, Xu B et al., Genome Biol; 2023, Akıncılar SC et al., Nucleic Acids Res; 2007, Kang Y et al., Oncogene). Therefore, we think the protein-protein interactions between CTCF and these TFs could contribute to the observed loop formation and regulation of mitosis genes in our study. We have added the information to the revised manuscript in the Results section. (****Line 363-368****)*

“… how many Sine elements are dysregulated in the mutant, is it possible to estimate this.”

***Response:*** *In Setdb1-KO NPCs, 12,179 SINE elements were upregulated, and 79 were downregulated in terms of CTCF binding (new* ***Fig. S4B****). We have added this information to the revised manuscript in the Results section. (****Line 256-257,*** ***260-261****)*

**Second round of review**

**Reviewer 1**

The author has addressed my concerns. There are some minor issues with the descriptions in the revised manuscript that need to be resolved before it can be accepted.

1. Abstract Section: Lines 43-44 lack coherence with the preceding and subsequent content. I suggest revising them for better flow.

2. The logic in the Discussion section is somewhat unclear. I suggest discussing SETDB1 before moving on to the discussion of SINE.

3. In the description of Cenpe in Figure 6, it is suggested to supplement its function, as well as its role in NPC, to aid in understanding.

**Reviewer 2**

The Authors addressed each comment from previous review, and added new figure panels and analyses as requested (mostly new integrative analyses of CTCF peaks with other epigenomic marks) and improved text and abstract

I do not have additional comments.

**Authors Response**

**Point-by-point responses to the reviewers’ comments:**

Point-by-point response to Reviewers
Reviewer #1:
1. “Abstract Section: Lines 43-44 lack coherence with the preceding and subsequent content. I suggest revising them for better flow.”
*Response: Thank you for the advice. As suggested, we haven rewritten the sentence for better coherence.*

2. “The logic in the Discussion section is somewhat unclear. I suggest discussing SETDB1 before moving on to the discussion of SINE.”
*Response: Thank you for the advice. As suggested, we have moved the discussion of SETDB1 ahead of the discussion of SINEs.*

3. “In the description of Cenpe in Figure 6, it is suggested to supplement its function, as well as its role in NPC, to aid in understanding.”
*Response: Thank you for the advice. As suggested, we have added the description of Cenpe in the Results section to explain its functions and roles in replicating cells including NPCs.*

Reviewer #2:
“The Authors addressed each comment from previous review, and added new figure panels and analyses as requested (mostly new integrative analyses of CTCF peaks with other epigenomic marks) and improved text and abstract.
I do not have additional comments.”
*Response: We thank you again for your helpful suggestions and appreciation of our study.*
